# Solar thermal polymerase chain reaction for smartphone-assisted molecular diagnostics

**DOI:** 10.1038/srep04137

**Published:** 2014-02-20

**Authors:** Li Jiang, Matthew Mancuso, Zhengda Lu, Gunkut Akar, Ethel Cesarman, David Erickson

**Affiliations:** 1 Sibley School of Mechanical and Aerospace Engineering, Cornell University, Ithaca, New York 14853; 2 Department of Biomedical Engineering, Cornell University, Ithaca, New York 14853; 3 School of Applied and Engineering Physics, Cornell University, Ithaca, New York 14853; 4 Department of Pathology and Laboratory Medicine, Weill Cornell Medical College, New York, NY 10065

## Abstract

Nucleic acid-based diagnostic techniques such as polymerase chain reaction (PCR) are used extensively in medical diagnostics due to their high sensitivity, specificity and quantification capability. In settings with limited infrastructure and unreliable electricity, however, access to such devices is often limited due to the highly specialized and energy-intensive nature of the thermal cycling process required for nucleic acid amplification. Here we integrate solar heating with microfluidics to eliminate thermal cycling power requirements as well as create a simple device infrastructure for PCR. Tests are completed in less than 30 min, and power consumption is reduced to 80 mW, enabling a standard 5.5 Wh iPhone battery to provide 70 h of power to this system. Additionally, we demonstrate a complete sample-to-answer diagnostic strategy by analyzing human skin biopsies infected with Kaposi's Sarcoma herpesvirus (KSHV/HHV-8) through the combination of solar thermal PCR, HotSHOT DNA extraction and smartphone-based fluorescence detection. We believe that exploiting the ubiquity of solar thermal energy as demonstrated here could facilitate broad availability of nucleic acid-based diagnostics in resource-limited areas.

Polymerase chain reaction (PCR)^1^ is widely used in nucleic acid-based diagnostics due to its high sensitivity, specificity and quantification capability^2^,^3^,^4^,^5^. Unfortunately, regions with minimal infrastructure and unreliable electricity often have limited access to such tools partly due to the relatively complex and energy-intensive nature of the thermal cycling process required for DNA amplification^3^,^4^,^5^. The need for diagnostic tests appropriate for point-of-care (POC) applications can be illustrated by Kaposi's Sarcoma (KS)^6^,^7^, a cancer caused by Kaposi's Sarcoma herpesvirus (KSHV). In sub-Saharan Africa, KS is associated with significant morbidity and mortality in adults and children^8^ and may be difficult to diagnose for several reasons. Various cutaneous lesions can clinically mimic KS, and overlapping histological features can make it challenging to distinguish KS from several other angioproliferative diseases^9^. In addition, serological tests for KSHV antibodies are unreliable because a KSHV infection is not necessarily sufficient for KS development, and over half of the population in endemic regions is serologically positive for this virus^7^. Similarly, performing viral detection in peripheral blood is inaccurate as not all KS patients have KSHV viremia while HIV+ patients without KS can exhibit KSHV viremia^10^. Performing PCR on viral DNA extracted from skin biopsies has demonstrated high sensitivity and specificity^6^, however high power and specialized equipment needs have been substantial obstacles against implementing PCR in POC settings.

Typically, PCR involves repeatedly cycling a sample through 95°C (denaturation), 60°C (annealing) and 72°C (extension) to achieve an exponential increase in target DNA. One of the lowest power devices was demonstrated by Wheeler *et al.*^11^, which expended 370 mW using convective PCR. Since then, works that have used smaller sample volumes or isothermal techniques have shown minimal improvement in power consumption. Additionally, such techniques often lead to trade-offs between simplicity, speed, reaction stability, throughput and power consumption^12^,^13^,^14^,^15^,^16^. Based on these works, a standard 5.5 Wh smartphone battery fully committed to heating such a system would last between 8 to 15 h. It is also important to note here that compared to these low power PCR techniques, both commercially available integrated nucleic acid-based devices as well as those found in recent literature typically require at least an order of magnitude more power. For example, the isothermal Gene-Z system developed by Stedtfeld *et al*.^17^ uses a lithium polymer battery that needs to be recharged after 4 h of operation. Based on the battery's power capacity, we estimate that the system consumes about 10 W during use. A 5.5 Wh smartphone battery would therefore enable 30 min of use before being depleted. Another example is the rapid PCR technique developed by Maltezos *et al*.^18^, which achieved ultrafast thermal cycling speed of about 3 s per cycle but consumed 400 W. A smartphone battery in this case would enable 1 min of use. In our system, solar thermal energy is utilized to drive nucleic acid amplification in a microfluidic chip. Using a smartphone for temperature sensing, the power consumption is reduced to 80 mW, representing a 2-orders of magnitude improvement compared to other state-of-the-art systems. An evaluation of techniques developed in the literature and commercially available devices is presented in Supplementary Information Table 1.

## Results

### Development and verification of solar thermal PCR

**Figure 1:**
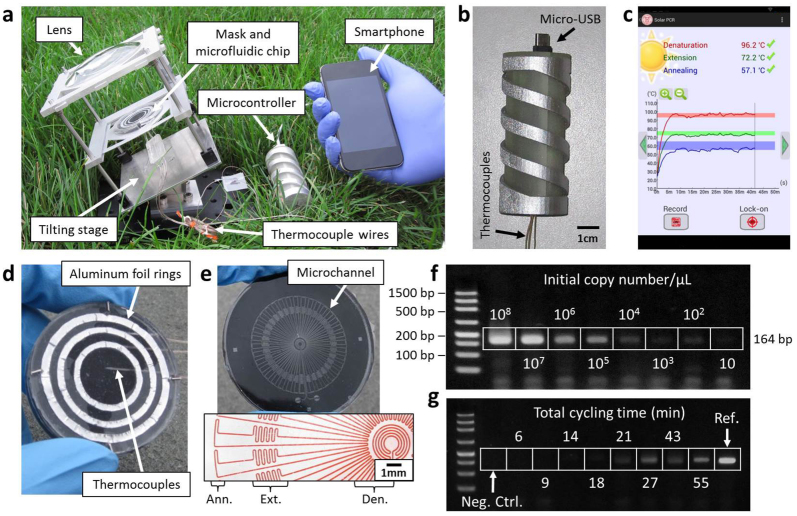
Solar thermal PCR system. (a), the benchtop component holds the lens, the PCR chip and a rotational stage. (b), Thermocouples on the chip are connected by a microcontroller to a smartphone. (c), a screenshot shows the app recording the temperatures over time. (d), the top of the PCR chip contains the mask and the thermocouples. (e), the bottom includes the absorber layer and the microfluidic channel, with marked regions for annealing, extension and denaturation (inset). (f), amplification over a range of concentrations. (g), amplification over a range of cycling speeds. The typical extension rate of *Taq* polymerase is 60–100 nucleotides/s at 72°C. Thus, 3 s should be sufficient for full extension of a 164 bp product. The design of our channel suggests that a minimum reaction time of 10 s/cycle is required. The band intensity began decreasing for reaction times faster than 20 s/cycle, and no band was observed for reactions faster than 10 s/cycle. Intensity values were normalized by a reference sample that was run in a conventional thermal cycler for 2 h.

With recent advancements in the field of optofluidics^19^, sunlight is finding a number of novel applications in both energy^20^,^21^ and global health^22^. Here we create solar thermal PCR, which eliminates the energy burden for nucleic acid amplification by employing sunlight to perform thermal cycling. Fig. 1a shows the device, which holds a 75 mm diameter glass lens and a movable 50 mm diameter PCR chip. These components are held on a 100° tilting stage, allowing the lens and chip to be positioned to face the sun throughout the day. Alignment between the chip and the sun is achieved by ensuring that the bright circle that the sun makes overlaps with the chip. Typically, the bright circle is several millimeters larger than the chip region that is heated, allowing for a relatively large tolerance in alignment. On the chip, the focused sunlight passes through a ring-shaped mask and is converted into heat by an absorber layer. Due to the masking of light in specific regions, three temperature zones at 95°C (denaturation), 72°C (extension) and 60°C (annealing) are created along the radius of the chip. A microfluidic channel then repeatedly guides a sample through these three zones for 35 cycles. The channel geometry dictates an approximate residence time ratio of 1:3:1 (denaturation:extension:annealing), effectively creating the thermal conditions that induce PCR. This microfluidic technique, known as continuous-flow PCR^23^,^24^, has exhibited fast reaction speeds^24^, minimal cross-contamination^25^, high throughput^26^, and facilitates microfluidic device integration^13^, making it highly attractive for POC applications. Three thermocouples in the chip are connected by a microcontroller (Fig. 1b) to a smartphone and a custom app measures the on-chip temperatures throughout the test (Fig. 1c and Extended Data Fig. 1). Above the thermocouples is the mask, comprised of three nested aluminum foil rings (Fig. 1d). Below the thermocouples is a disposable piece containing the light absorber and the microfluidic channel (Fig. 1e). The absorber is made from a carbon black and PDMS mixture, which was shown previously to allow no light transmission within the visible spectrum at the same concentration and thickness as the present work^27^. In addition, carbon black powder was shown to have excellent broadband absorption^28^ across the spectrum. We therefore assume the solar thermal energy conversion of the carbon black itself is nearly 100% efficient because the vast majority of the absorbed light energy is necessarily converted into heat. To characterize the effect of the PDMS on the absorber's photo-thermal efficiency, we measured the intensity of sunlight after passing through a clear 1 mm thin PDMS film using a powermeter. The 1 mm PDMS thickness is consistent with the thickness of the PDMS above the carbon black layer. The power lost through the film was measured to be 5%. This is supported by Cai et al.^29^, which showed excellent transmittance of PDMS below 1200 nm. The entire absorber therefore is estimated to be about 90% to 95% efficient. Details on device fabrication are available in Supplementary Information.

To demonstrate solar thermal PCR in the range of KSHV DNA counts expected from a punch biopsy, we amplified plasmid samples with starting DNA concentrations ranging from 10^8^ to 10 copies/μL, shown in Fig. 1f. A 164-base pair (bp) segment of the KSHV gene vCyclin was selected as the target because the sequence is unique and conserved among different strains (Supplementary Information Table 2). Bands appeared for all samples when analyzed by gel electrophoresis (Supplementary Information Fig. 2). PCR dependence on flow rate was also analyzed to determine the fastest speed with which a test can be performed. We conducted experiments using 10 μL samples with cycling times ranging from 5 s/cycle to 50 s/cycle. This range corresponded to total reaction times of 6 min to 55 min (Fig. 1g), which was defined as the time taken between when the front end of the sample enters the chip and when the back end exits the chip. Band intensity increased significantly near 20 s/cycle (Extended Data Fig. 2), showing that a 10 μL sample can be amplified and extracted within 30 min. Details on DNA count estimation and plasmid extraction are available in Supplementary Information.

### Demonstration of solar thermal PCR under a variety of ambient conditions

**Figure 2:**
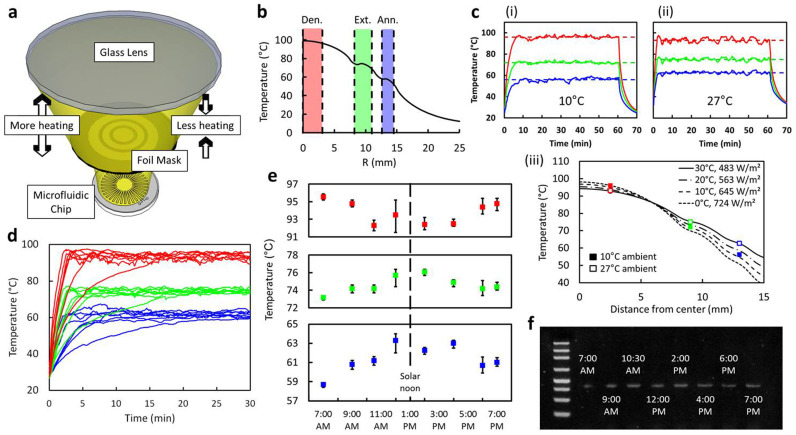
Thermal characterization and demonstration of solar thermal PCR. (a), schematic showing the ability to change the solar intensity by changing the lens-to-chip distance. (b), a simulation showing the plateaued temperature profile at the plane of the microchannel. (c), measurements in (i) April and (ii) May demonstrate the ability to achieve similar on-chip temperatures using the same mask. (iii) Simulations provide thermal profiles from 0°C to 30°C ambient temperature. (d), Thermal measurements from 7 AM to 7 PM show relatively consistent values. (e), averaged temperature data shows day-long trend. f, PCR tests show that hourly changes in ambient temperature still allowed for product amplification. For Fig. 2c, d, and e, the red, green, and blue color curves respectively refer to the denaturation, extension, and annealing temperatures measured in the app.

Although changes in solar intensity and ambient temperature can affect on-chip temperatures, we mitigate the issue by simply adjusting the distance between the lens and the chip, illustrated in Fig. 2a. This adjustment is done manually based on temperature feedback provided by the app. Changing the lens-to-chip distance in turn changes the intensity of the sunlight absorbed by the chip, which compensates for different ambient temperatures and allows the system to function under a range of conditions. Based on simulations (Supplementary Information Fig. 3), we designed the masking rings such that the thermal profile generated by the masked light exhibits plateaus near 95°C, 72°C and 60°C for a given solar intensity and ambient temperature (Fig. 2b). The chip can then be lowered to increase solar heating on cooler days and raised to reduce heating on warmer days. Fig. 2c shows temperatures obtained in April (10°C ambient) and May (27°C ambient) of 2013 in Ithaca, NY using the same mask. For these measurements, the lens-to-chip distance was first set to 85 mm to quickly heat the chip. Once temperatures near PCR requirements were reached, the distance was reduced to 79 mm in April or 68 mm in May. Through this process, on-chip temperature changes were minimized to 3°C for denaturation and extension and 6°C for annealing. Simulations suggest that over the range of 0°C to 30°C roughly 75% to 50% of peak insolation (1000 W/m^2^) is sufficient for PCR (Fig. 2c (iii)).

After having developed the solar thermal PCR system to work under a range of conditions, we demonstrated that PCR can be performed for approximately 12 h each day during the summer months. Fig. 2d shows on-chip temperatures in July from 7 AM to 7 PM. By setting the lens-to-chip distance at 85 mm, the necessary temperatures were usually obtained within 3 min, while longer times were required in the morning when ambient temperature is cooler and sunlight is less intense. For the data presented, temperatures varied from 25°C in the morning to 32°C in the early afternoon. Fig. 2e shows that as the day warmed in the morning, the denaturation temperature decreased while the extension and annealing temperatures increased. The trends were reversed in the late afternoon as ambient temperature cooled. For these experiments, the lens-to-chip distance and the tilt were both readjusted for each new test, however no adjustment was required within each test after PCR temperatures were reached. In practice, a set of different masks can be provided to the user, each having been optimized for a specific temperature range. Fig. 2f demonstrates that PCR can be successfully performed for most of the daylight hours, although larger thermal fluctuations, particular for tests at 12:00 PM and 6:00 PM, may have caused reduced amplification efficiency, as shown by the decrease in band intensity (see Supplementary Information Fig. 4 and Fig. 5).

**Figure 3:**
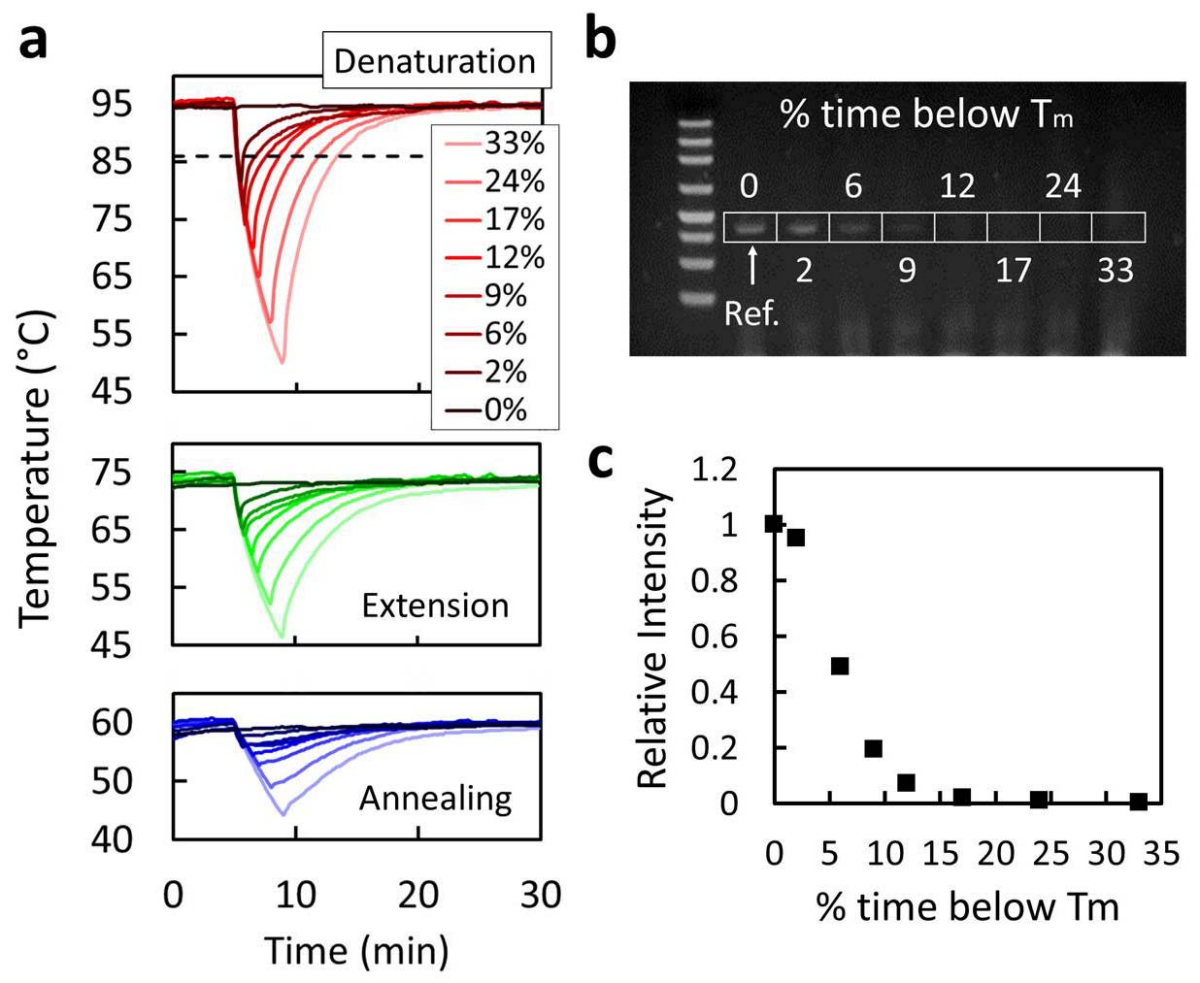
Characterization of simulated cloud coverage and flow rate. (a), temperature variation for denaturation, extension and annealing due to blocking of the light. Simulated clouding times ranged from 0 s (darkest curve) to 4 min (lightest curve) for a total test time of 27 min. The percentages represent the percent of time that each test spent in non-ideal PCR conditions, which for these tests was defined as when the denaturation temperature dropped below the dashed line. (b), gel electrophoresis and (c), corresponding band measurements show diminishing intensities as a function of the duration of simulated cloud coverage.

In the field, clouds could manifest in a number of forms that affect PCR efficiency. To examine the influence of clouding in a controlled manner, we designed a solar simulator using a 100 W LED. Optical lenses were set up to collimate the light and create similar temperatures on the chip. To mimic clouding, the light was blocked 5 min after the PCR process began for a duration that ranged from 15 s to 4 min. The resulting thermal profiles are shown in Fig. 3a. The DNA melting temperature of 86°C, calculated using the nearest neighbor method, served as a threshold for the denaturation step to define the percent of time that the sample spends below acceptable conditions for PCR. These were calculated to range from 2% (15 s light obstruction) to 33% (4 min light obstruction) for tests with a total flow-through time of 27 min. The band intensities shown in Fig. 3b, c suggest an exponential decay as the duration of simulated clouding increases. Details on the LED setup are available in Supplementary Information.

### Sample-to-answer analysis of human skin biopsies

**Figure 4:**
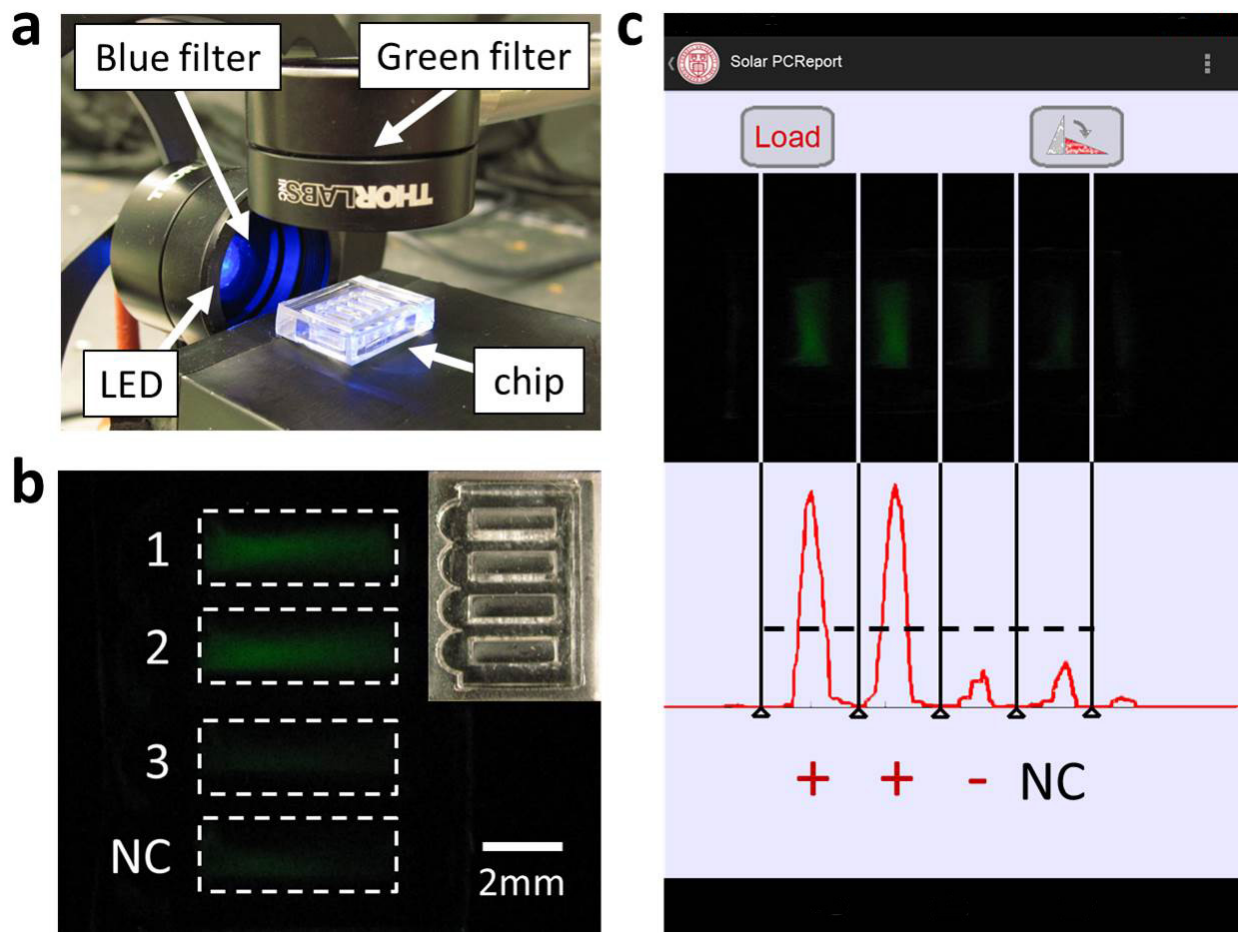
Integration of HotSHOT cell lysis, solar thermal PCR and smartphone-based detection applied to mouse tail biopsies. (a), fluorescence detection setup includes a blue filter between the LED and the chip and a green filter between the chip and the camera. (b), PDMS chip (inset) containing 4 tests: solar thermal PCR performed using KSHV+ samples (1, 2) and a KSHV- sample (3) and traditional PCR using negative control (NC). (c), a screenshot of the app that analyzes the fluorescence signals, showing high intensities for samples 1 and 2 and low intensities for sample 3 and NC.

To demonstrate compatibility with solid tissue processing in the absence of specialized equipment, we analyzed human skin biopsies both with and without KS involvement by combining solar thermal PCR with single-tube HotSHOT^30^ DNA extraction and smartphone fluorescence detection. Fig. 4a shows a smartphone-powered blue LED incident on a PDMS chip containing 4 samples. Each sample includes SYBR Green dye, which preferentially binds to double-stranded DNA and emits green light when excited by blue light. The chip shown in Fig. 4a, b contains samples processed from two KS biopsies (1, 2), a skin biopsy with mycosis fungoides but without KS (3), and a negative control (NC). Samples 1 – 3 were mixed with dry room-temperature PCR reagents and primers and amplified by solar thermal PCR, while NC was mixed with a conventional refrigerated PCR reagent kit and run in a thermal cycler. An app compared the average fluorescent signals of the three test samples to NC, providing the user with the correct diagnosis for each (Fig. 4c). In practice, an intensity threshold could be determined based on multiple tests to provide on-site diagnosis. By tracking the battery depletion of the smartphone over a number of PCR tests, we calculate a power consumption of 80 mW, which is two orders of magnitude lower than commercial products (Supplementary Information Table 1). For a 5.5 Wh smartphone battery, this would enable a battery life of 70 h, compared to about 15 h for techniques in literature and 1 h for commercial devices.

## Discussion

Solar thermal PCR as described here supports a number of qualities beneficial to POC nucleic acid based diagnostics. Sunlight-driven DNA amplification removes the power requirements for thermal cycling and enables a 100-fold reduction in power consumption compared to state-of-the-art devices, allowing it to be powered by a smartphone battery for 70 h. Tests can be performed in less than 30 min, potentially enabling rapid diagnostics in regions where long travel distances to clinics make follow-up meetings with patients difficult. The system is also highly efficient in energy conversion and supports minimal infrastructure by removing a number of electrical components such as microheaters and actuators. We demonstrated DNA amplification under a range of ambient conditions and successfully analyzed human skin biopsies by combining solar thermal PCR with HotSHOT DNA extraction and smartphone-based detection. Compared to battery or solar panel-powered systems, solar thermal PCR offers advantages in power consumption and simplicity without compromising PCR performance. Such a strategy could be developed into an integrated device completely powered by sunlight and a smartphone, leading to greater accessibility of DNA diagnostics in resource-limited settings.

## Methods

### PCR sample preparation

A 70 μL volume of DI water containing 4.3% w/v polyvinylpyrrolidone (PVP) (Sigma-Aldrich, 437190) is mixed with PCR reagents (Invitrogen, N8010055) including 10 μL of 10X PCR buffer, 0.2-mM dNTPs, 10 Units/100 μL of *Taq* polymerase, 1 μM of forward and reverse primers and 1 μL of target DNA. PVP is used here to inhibit *Taq* adsorption onto the PDMS surface^24^,^31^.

### Solar thermal PCR procedure

The channel was first passivated with a 7.5 mg/mL bovine serum albumin (Sigma-Aldrich, A7888) solution for 2 h to further inhibit *Taq* adsorption, and flushed with DI water at 1 μL/min for 30 min to remove unbound particles. During the test, a 20 μL paraffin oil plug (VWR, BDH3338) was pumped through the chip, followed by a 10 μL sample, and then another oil plug. The two plugs prevent sample evaporation caused by heating^32^.

Unless specified, samples contained an initial DNA concentration of 10^5^ copies/μL and tests were conducted with a syringe pump (New Era, NE-1000) set at 1 μL/min. Due to absorption of the oil into the PDMS, the actual flow rate was calculated to be 0.8 μL/min based on the time the samples took to go from the inlet to the outlet.

### Sample preparation for smartphone fluorescence detection

The negative control included 25 μL of *Power* SYBR Green PCR master mix (Invitrogen, 4368577) combined with 15 μL of DI water, 1 μM of forward and reverse primers and 10 μL of KSHV- solution. Tests 1–3 used High Yield PCR EcoDry Premix (Clontech, 639278) mixed with 15 μL of 5% w/v PVP in DI water, 10 Units/100 μL of *Taq* polymerase, 1 μM of forward and reverse primers, and 10 μL of KSHV+ biopsy solution (samples 1 and 2) or KSHV- solution (sample 3). After amplification, 10 μL of the products were added to 10 μL SYBR Green solution and injected into the chambers.

## Author Contributions

L.J., E.C., and D.E. conceived the project. L.J. designed and performed the experiments and analyzed the data. L.J. and Z.L. designed the electronics packaging. Z.L. and M.M. designed the smartphone apps. M.M. selected and designed the DNA sequence and primers for PCR. E.C. and G.A. provided the plasmid DNA and the KSHV+ and KSHV- cell lines. G.A. performed the HotSHOT DNA extraction. The paper was written and edited by L.J., E.C. and D.E.

## Supplementary Material

Supplementary Information Supplementary Info
