# Supplementary material for: Solar thermal polymerase chain reaction for smartphone-assisted molecular diagnostics
Source: Sci Rep. 2014 Feb 20;4:4137. doi: 10.1038/srep04137 (PMC3929917; doi:10.1038/srep04137)
Supplement: Supplementary Information — Supplementary Info [file srep04137-s1.pdf]

Supplementary Information for:

Solar thermal polymerase chain reaction for smartphone-assisted molecular diagnostics

Li Jiang[1], Matthew Mancuso[2], Zhengda Lu[3], Gunkut Akar[4], Ethel Cesarman[4] and David Erickson[1*]

[1]*Sibley School of Mechanical and Aerospace Engineering, Cornell University, Ithaca, New York 14853*

[2]*Department of Biomedical Engineering, Cornell University, Ithaca, New York 14853*

[3]*School of Applied and Engineering Physics, Cornell University, Ithaca, New York 14853*

[4]*Department of Pathology and Laboratory Medicine, Weill Cornell Medical College, New York, NY 10065*

*To whom correspondence should be addressed. Email: de54@cornell.edu

Table 1. Projected battery life of PCR devices using smartphone/tablet battery

Paper/device	Sample volume (µL)	Power Consumption (mW)	# of samples	Sample length (bp)	Battery life (h)		
					iPhone 5[10]	Galaxy S4[11]	Nexus 7[12]
**Academic works, power consumption assessed for heating only**							
PCR amplification (multiple temperatures)							
Liu *et al.*[1]	0.012	460	1	199	12	21	35
Krishnan *et al.*[2]	15	360	1	191	15	27	44
Wheeler *et al.*[3]	75	370	1	90	15	27	44
Neuzil *et al.*[4]	20	3,000	1	-	2	3	5
Isothermal amplification							
Jenkins *et al.*[5]	25	700	1	-	8	14	23
Smith *et al.*[6]	30	450	1	-	12	21	35
**Commercial devices/Pipeline products**							
Palm PCR F1-12[7] (Ahram Biosystems, Inc.)	20	5,000	<12	< 2000	1	2	3
*Liat analyzer[8,9] (IQuum, Inc.)	10 - 50	10,000	1	-	0.5	1	1.5
Solar thermal PCR	10	App: 50; Stand-by: 24[10], 27[11], 53[12]	1	164	74	128	155

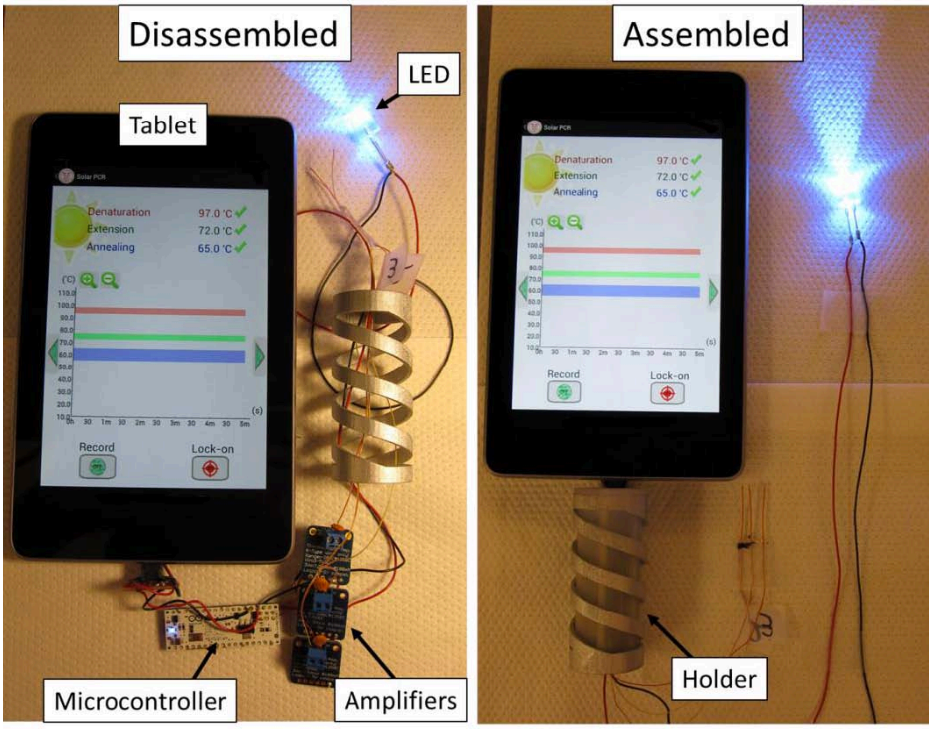

Fig. 1. Disassembled view of electronic components. The electronics include an Arduino Micro microcontroller and three thermocouples amplifiers. A tablet is shown here to power the thermocouple readings during PCR and light a 3.4 W LED for fluorescent detection.

Table 2. Probe and Target Sequences for KSHV

Name	Sequence
KSHV Probe 1 (22 bp)	AGGATCTGTTCCACTGCCGCCT
KSHV Probe 2 (22 bp)	ACTGGCTCATTGCCCGCCTCTA
KSHV Target (164 bp)	AGGATCTGTTCCACTGCCGCCTGTAGAACGGAAACATCGCATCCCAATATGCTTGC<br>CAGCTGAGGAACTACCCCACCCGAGTGGGTATCCTGCGGAATGACGTTGGCAGG<br>AACCAACAGCGCACAGCCTGCAGCGCTGATAATAGAGGCGGGCAATGAGCCAGT

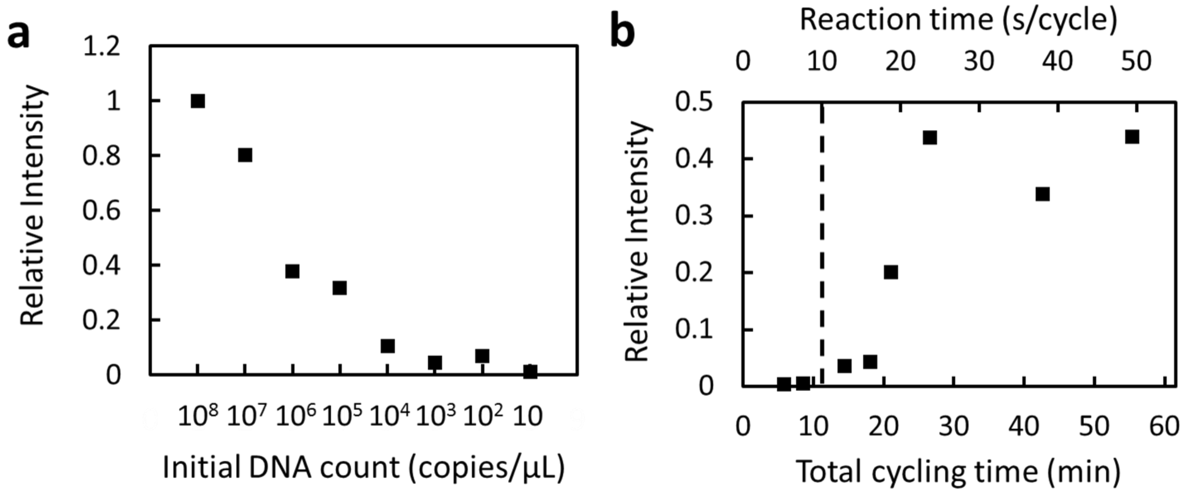

Fig. 2. Measurements of fluorescence intensities. a, data for varying DNA concentrations corresponds to Fig. 1f in paper. **b**, data for flow rate corresponds to Fig. 1g in paper. Dashed line represents the theoretical minimum cycling speed for the extension step.

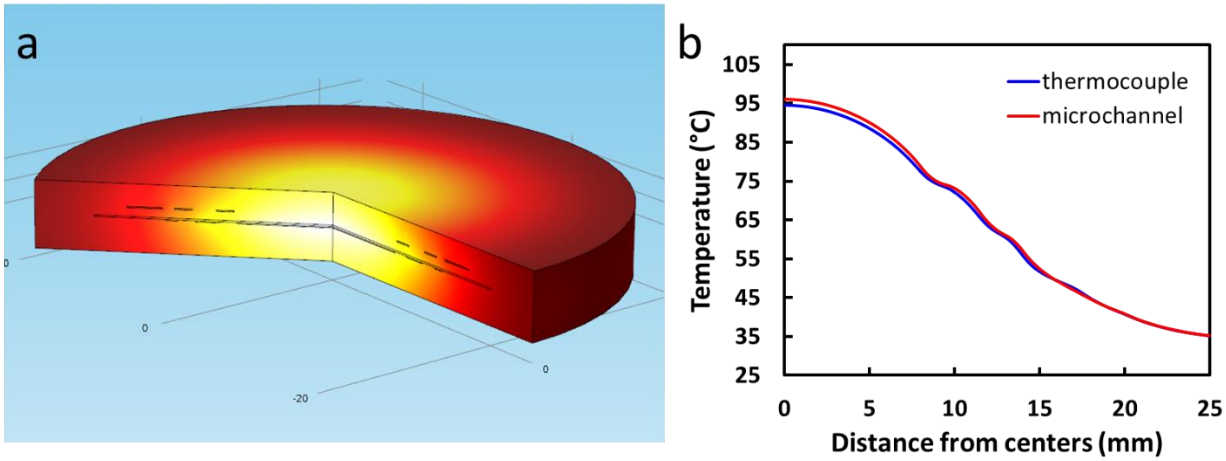

Fig. 3. Comsol simulation of solar thermal PCR chip. a, heat transfer model includes the PDMS, foil mask and glass cover slip. **b**, in our design the thermocouples are 500 µm above the absorber, while the microfluidic channel is 250 µm below the absorber. The plot of temperature values at these two locations show little different, suggesting that thermocouple measurements are adequate for temperature sensing in the channel.

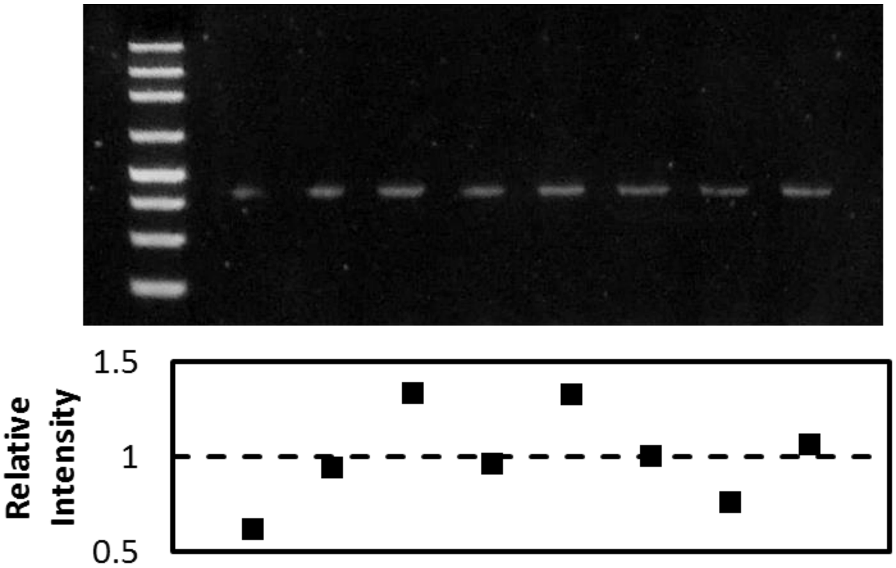

Fig. 4. Analysis of band intensity versus time of day . Gel image is the same as Fig. 2f. Relative band intensities were analyzed using imageJ, with a value of 1 set as the average brightness. It is difficult to discern any particular trend from these tests, although it may be important to note that tests 4 (12:00 PM) and 7 (6:00 PM) could be less bright due to the higher thermal fluctuation associated with those tests, as shown by the larger standard deviation displayed in Fig. 2e.

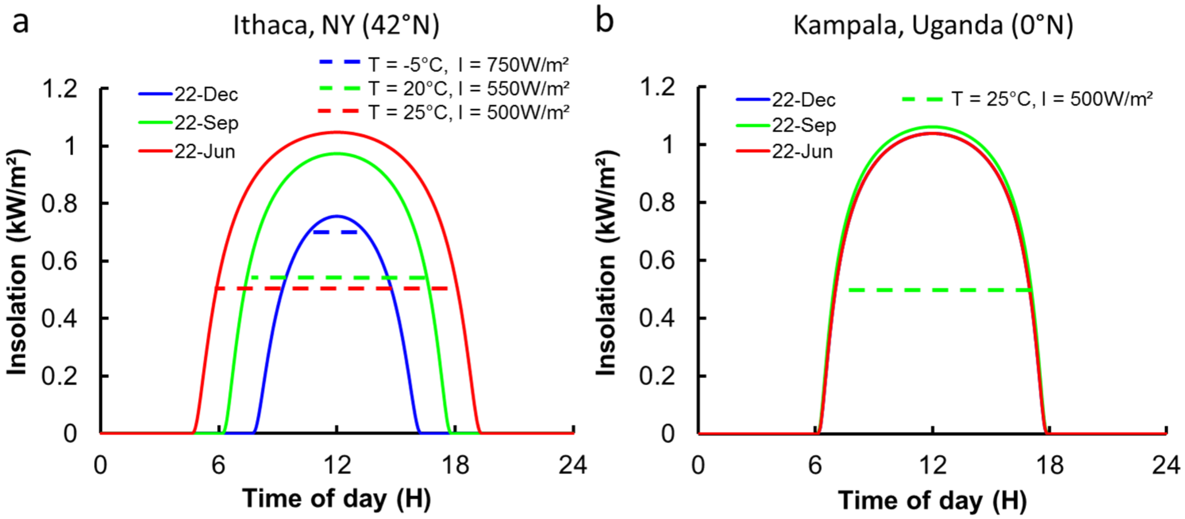

Fig. 5. Availability of sunlight compared to PCR requirements. Insolation values were calculated for the winter solstice (Dec. 22), equinox (Sep. 22) and summer solstice (Jun. 22) for Ithaca, NY (42°N) and Kampala, Uganda (0°N). The average temperatures during each period were used to estimate the minimum solar intensity required for thermal cycling. **a,** in the summer in Ithaca, solar thermal PCR can be powered for about 12 h each day. **b,** in Uganda, where insolation values are much more uniform, solar thermal PCR can be powered for 10 h each day throughout the year.

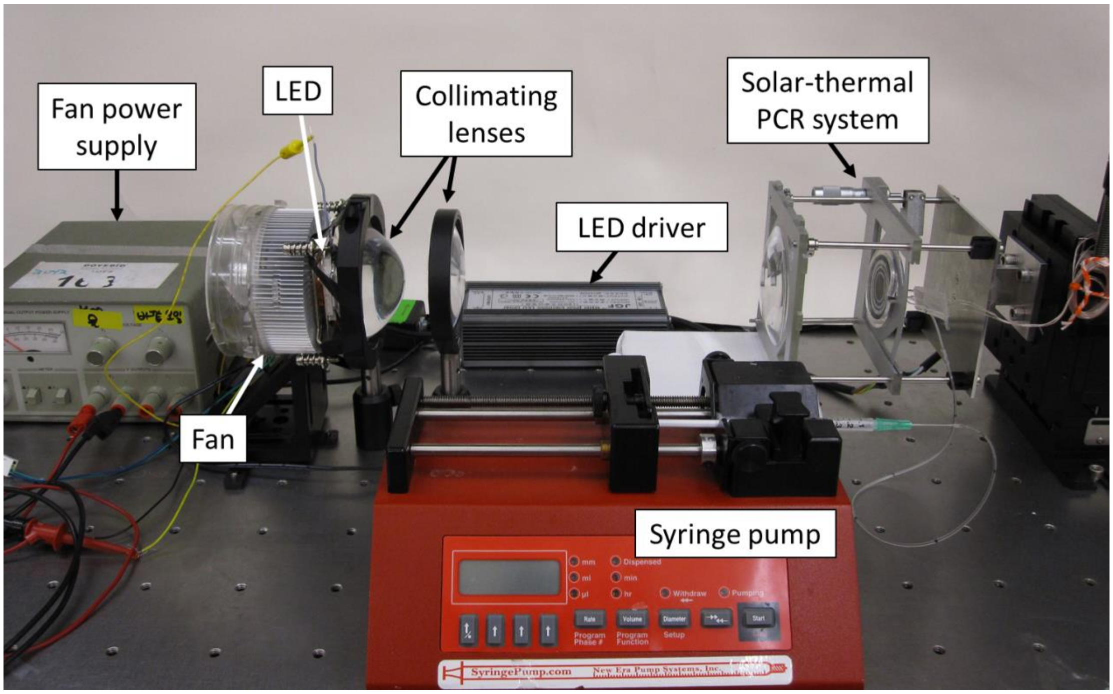

Fig. 6. Solar simulator. The setup includes a 100 W white LED, a cooling fan and aluminum heat sink, and collimating lenses to simulate solar illumination in the lab.

Benchtop system design

The platforms holding the lens and the microfluidic chip were designed in AutoCAD and 3D-printed (Stratasys, Object500 Connex). The bottom hinge, which allows for 100° rotation of the stage, was machine shopped at Cornell's Laboratory of Atomic and Solid State Physics (LASSP). The assembled system includes: 1x 75 mm lens (Thorlabs, LA1002), 4x cage assembly rod (Thorlabs, SR4), 4x cage assembly rod (Thorlabs, SR05), 4x rod adapter (Thorlabs, SRSCA), 4x thread adapter (Thorlabs, MSA8), 1x breadboard (Thorlabs, MB1015/M), 1x cage rod cross coupler (Thorlabs, S1A), and 1x micrometer head (Thorlabs, 150-801ST).

Microfluidic chip design

The PCR chip is 50 mm in diameter and 5 mm in thickness and is comprised of three parts. The top part holds three thermocouples (Omega, 5TC-TT-K-36-36) placed immediately below the three aluminum foil rings. These are secured in place inside 2 mm of PDMS (10:1 ratio of base to curing agent). The bottom piece contains the absorber layer and the microfluidic channel. The absorber is a mixture of carbon black, PDMS base and PDMS curing agent in a 0.02:1:0.05 weight ratio. This is spin-coated onto a 40 mm diameter glass cover slip (Warner Instruments, 64-1500) at 1800 rpm to achieve a film thickness of 100 µm. A clear 50 µm thick PDMS layer is spin-coated above the carbon black layer to prevent any potential interactions between PCR reagents in the channel and the carbon black. The microfluidic channel, formed using standard photolithography technique, is plasma bonded to the PDMS above the absorber to enclose the channel. The microfluidic piece is then placed with the top section containing the mask and thermocouples. At this point, the chip is 5 mm thick and 40 mm in diameter. A final 10 mm thick PDMS ring is wrapped around the chip, securing it to the benchtop platform and also providing additional insulation. Because these three pieces are all separate, the microfluidic section is easily replaced after each test.

To make the microfluidic channel, SU-8 2075 photoresist (Microchem) is spun on a silicon wafer at 2200 rpm for 30 s to obtain a 100 µm thick film. The SU-8 is then patterned through a chrome photomask (designed in L-Edit, written with the Heidelberg Mask Writer DWL2000)

using an ABM Contact Aligner. Uncured SU-8 is removed using SU-8 developer. PDMS is poured over the SU-8 master to a thickness of 3 mm and cured at 80°C for 2 h. The channel cross section is 100 µm × 100 µm and is 1.2 m in length. As a fluid element passes through the channel, it first encounters an initialization zone near 95°C at the center of the chip of approximately 30 mm in length. It then passes through 35 cycles of the denaturation, annealing and extension zones. Each cycle corresponds to a 32 mm long channel section, which radially spans 10° of space, adding up to 350°. The last 10° is dedicated to a final extension step before the fluid exits.

Microcontroller and app design

The microcontroller includes an Arduino Micro board base (Nexuscyber Electronics, ATmega32u4) and three thermocouple breakout amplifiers (Adafruit Industries, MAX31855K) which are each connected with a K-type thermocouple from the chip. To reduce the noise, a 0.01µf capacitor (AllElectronics Corporation, 103D50) is added across each thermocouple lead. Among the 8 pins of the breakout amplifiers, T+ and T- are used to detect slight changes in voltage between thermocouple leads, while the rest process the reading from the thermocouple and transmit the data through a serial interface. The Arduino Micro reads the serial data from the amplifiers and output it to the smartphone.

The Android app is developed in Eclipse with the plug-in Android Development Tools (ADT) and Android SDK. Coding with Arduino 1.0.5 is required for serial data transmission between the MAX31855K amplifier and the Arduino Micro.

KSHV target selection and primer design

Within the KSHV genome, part of the DNA that codes for vCyclin was chosen as our target sequence. vCyclin and its associated RNA are expressed during both the latent and lytic viral phases[13]. Specific primers for this sequence were chosen using BLAST Primer Design[14], and ordered from Invitrogen (Grand Island, NY). Sequence information can be found in Extended Data Table 2.

DNA count estimation and plasmid culturing and extraction

For Kaposi's sarcoma, the KSHV DNA count varies from case to case depending on the amount of connective tissue versus cells acquired in a skin biopsy. Approximately 10^6 cells can be obtained in a 3 mm punch biopsy of skin lesions, and we estimate that on average there is 1 copy of KSHV DNA per cell. Therefore, we assume a typical biopsy to contain 10^6 copies of KSHV DNA.

With the exception of the skin biopsies used in Fig. 4 of the paper, all tests were performed using vCyclin plasmid. In order to get multiple copy of vCyclin plasmid, MAX Efficiency DH5α Competent Cells (Invitrogen, 18258-012) are used for transformation. The transformation procedure is as follows: 1) Thaw competent cells on wet ice. 2) Gently mix cells, then aliquot 100 µL into chilled Eppendorf tubes. 3) Add 1 µL of plasmid to the cells, gently mix by pipetting up and down. 4) Incubate the cells on ice for 30 min. 5) Heat-shock cells for 45 s in a 42°C water bath. 6) Place the cells on ice for 2 min. 7) Add 0.9 mL room temperature SOC Medium (Invitrogen, 15544-034). 8) Shake at 225 rpm, 37°C for 1 h. 9) Spread 50 to 100 µL of this culture on LB plates with 100 µg/mL ampicilin. 10) Incubate overnight at 37°C. After overnight incubation single colonies are picked up and inoculated in 3mL of LB medium containing ampicilin with vigorous shaking at 37°C for 14 to 16 h. After the incubation DNA is extracted using QIAprep Spin Miniprep Kit (QIAGEN, 27106).

Thermodynamics modeling

COMSOL Multiphysics was used to create a 2D rectangular model of the heat transfer inside the PCR chip (Extended Data Fig. 3). Inside the system, we solved for the steady state heat transfer equation

$$0 = \vec{\nabla} \cdot k\vec{\nabla}T + Q \qquad (1)$$

where k is the thermal conductivity, T is the temperature and Q is the volumetric heat flux. Axial symmetric boundary condition was applied on the left side, corresponding to r = 0. On the top, bottom and ride sides we applied convective and radiation boundary conditions

$$\boldsymbol{n} \cdot \left(-k\vec{\nabla}T\right) = h(T - T_{ext}) + \varepsilon\sigma(T^4 - T_{ext}^4) \qquad (2)$$

Where h is the heat transfer coefficient, T_{ext} is the external temperature, ε is the emissivity, and σ is the Stefan-Boltzmann coefficient.

In the model, this was simplified as three ring-shaped heat sources placed at the same locations as where light passes through the foil rings mask. The placement and dimensions of the rings can be adjusted to support the range of ambient temperatures that is expected for the test. In our design, the rings were placed at $8 \leq r \leq 9.5;\ 11.5 \leq r \leq 13; 14 \leq r \leq 17$, where r is the radius in mm. In the model, this corresponds to heat sources placed at $0 \leq r \leq 8;\ 9.5 \leq r \leq 11.5; 13 \leq r \leq 14$. Using the Beer-Lambert law, we calculated the volumetric heat flux Q generated through the absorber, which is given by

$$Q = \alpha I_f e^{-\alpha z} \qquad (3)$$

where α is the absorption coefficient, I_f is the focused light intensity incident on the chip, and z is the depth into the absorber. The absorption coefficient for the PDMS-carbon black mixture was previously calculated[15] to be 0.18 μm^{-1}. The incident light intensity I_f is calculated using

$$I_f = I_0 \left(\frac{1}{1-L_c/f}\right)^2 \qquad (4)$$

where I_0 is the unfocused solar intensity, L_c is the distance between the lens and the chip, and f is the focal length of the lens.

The thermocouples and the microchannel are assumed to have negligible effect on the heat transfer in the chip due to their relatively small size compared to the bulk of the system.

LED solar simulator setup

Extended Data Fig. 5 shows the solar simulator setup. The system includes a kit containing a 100 W white LED, AC driver and lens (Topledlight, BY-HP100WWH, BY-DR100WW) and a cooling fan aluminum heatsink (Topledlight, BY-HS100). We found that the provided lens did not fully collimate the light, and therefore added two additional lenses (Thorlabs, LA1740, LA1238) at 7 cm in front of the LED. The solar thermal PCR system was secured sideways on a translation stage (Thorlabs, LT3) and placed at 20 cm beyond the additional lenses.

Method for power consumption calculation and comparison to state-of-the-art

For characterizing the power consumption we ran experiments using a Galaxy S4 smartphone (9.88 Wh battery capacity) and a Nexus 7 tablet (16 Wh battery capacity). In Extended Data Table 1, these battery capacities are divided by the power consumption of several academic works and commercial devices to calculate the projected battery life.

We calculated power consumption in our system based on the percentage of battery drained over a series of tests. During these tests the app is opened periodically to monitor the temperatures. On average, for each test (1 sample, 30 min) this resulted in a total of 3 min of screen time to include thermal measurements and fluorescent detection and 27 min in stand-by. Tests were repeated until 5% of the battery for each device was consumed. For the Galaxy S4, this took about 12 tests (6 h), whereas the Nexus 7 required 15 tests (7.5 h). By dividing the amount of energy used over the time period, we arrived at power consumptions of 80 mW for the Galaxy S4 and 100 mW for the Nexus 7. The maximum number of hours on stand-by reported for the devices is 370 h for the Galaxy S4 and 300 h for the Nexus 7. Thus, we calculate stand-by power consumption values of 27 mW for the Galaxy S4 and 53 mW for the Nexus 7. Therefore, the microcontroller and screen collectively consume around 50 mW, enabling 128 h and 155 h of battery life for the galaxy S4 and the Nexus 7, respectively. The 50 mW power consumption of the app was used to calculate a 74 h battery life for an iPhone 5 (5.45 Wh battery capacity, 24 mW standby power consumption).

The above calculating was performed for one test, however there are advantages to how our system would scale with the number of tests as well. One consideration is in running parallel tests using multiple devices. For traditional machines the power consumption would scale linearly with the number of devices. The advantage of our technique is that one smartphone/tablet can be connected to different PCR systems to measure the temperature. This would mean that if the app is being used continuously for different devices, the stand-by power consumption could be eliminated and that each test may require as little as 50mW. The other way to analyze power requirements is with running multiple tests in one chip. Here our

system differs from other devices in that the samples would be run in series within the same channel instead of in parallel in multiple tubes/wells. The power consumption would remain the same but the total energy required would increase with the number of samples because it would take longer for the samples to pass through the chip. Specifically, the energy consumption would scale linearly with the testing time, and therefore the exact amount of energy required would depend on the sample volume. It may be possible to adapt our system to perform high throughput PCR with small sample volumes, in which case running a number of samples would require a minimal increase in energy.

There is some difficulty in comparing power consumption of our device to that of other systems, with the issue being that the powers reported for the academic works or devices are not applied to perform the same functions. For example, most of the academic works report power consumption for heating alone. The Palm PCR reports power consumption required for PCR, which includes heating and mechanical actuation within the device. In our system we do not need to supply any power for heating, and therefore a strict comparison of heating alone would mean that we completely eliminate power requirements. The 80mW reported in our device includes PCR and fluorescence detection. In addition, although the HotSHOT sample processing was performed using a heater for the experiments in this paper, it only requires heating and could foreseeably also be powered by sunlight – something that we are currently developing. Therefore, one of the advantages of our technique is that the entire sample-to-answer process can be predominately performed by solar thermal energy. For these reasons, we believe the order of magnitude comparison between our system and bench top devices such as the Liat Analyzer is a more appropriate, albeit for now imperfect, analysis.

HotSHOT DNA isolation from tissue samples

The HotSHOT procedure includes two reagents. The first is an alkaline lysis reagent with 25mM NaOH, 0.2 mM disodium EDTA at a pH of 12 prepared by dissolving the salts in water without adjusting the pH. The second is a neutralizing reagent with 40 mM Tris-HCl at a pH of 5 prepared by dissolving Tris-HCl in water without adjusting the pH.

KS and control tissue samples were archival, from the Pathology Laboratory of Weill Cornell Medical College/The New York Presbyterian Hospital, and obtained with proper institutional review board approval. These were stored as fresh-frozen specimens in OCT embedding media. Frozen sections were used to determine involvement by KS by histological examination of an H&E stained slide as well as by immunohistochemistry for KSHV LANA. The specimens used in Fig. 4 of the text were skin punch biopsies (3 mm), and the negative control was a skin biopsy with mycosis fungoides but without KS. After collecting the samples in a tube, 75 µL alkaline lysis reagent is added to the samples and heated to 95°C for 30 min. After heating, samples are cooled to 4°C, and 75 µL neutralizing reagent is added to each sample. Following this, 10 µL of the final preparation was mixed with the respective PCR reagents, as detailed in Methods Summary.

Smartphone fluorescent detection setup

A PDMS chip containing 4 chambers was designed to each hold 20 µL volumes of the samples. A cylindrical lens 2 mm in diameter was molded in the PDMS in front of each chamber to focus the incident light. A 3.4 W blue LED (Sparkfun Electronics, COM-08860) was used as the light source. A blue filter (Thorlabs, FGB25) was placed in front of the LED to reduce light at undesired wavelengths. A green filter (Thorlabs FGV9) is placed above the PDMS chip, perpendicular to the direction that the LED excites the samples. An image of the excited samples is taken in the dark through the green filter. The app then plots the fluorescent signal intensity across each sample, resulting in high peaks for the KSHV+ samples and low peaks for the KSHV- sample and the negative control.

References

1 Liu, J., Enzelberger, M. & Quake, S. A nanoliter rotary device for polymerase chain reaction. *Electrophoresis* **23**, 1531-1536 (2002).

2 Krishnan, N., Agrawal, N., Burns, M. A. & Ugaz, V. M. Reactions and fluidics in miniaturized natural convection systems. *Anal. Chem.* **76**, 6254-6265 (2004).

3 Wheeler, E. K. *et al.* Convectively driven polymerase chain reaction thermal cycler. *Anal. Chem.* **76**, 4011-4016 (2004).

4 Neuzil, P. *et al.* Rapid detection of viral RNA by a pocket-size real-time PCR system. *Lab Chip* **10**, 2632-2634 (2010).
5 Jenkins, D. M., Kubota, R., Dong, J., Li, Y. & Higashiguchi, D. Handheld device for real-time, quantitative, LAMP-based detection of Salmonella enterica using assimilating probes. *Biosens. Bioelectron.* **30**, 255-260 (2011).
6 Smith, M. C., Steimle, G., Ivanov, S., Holly, M. & Fries, D. P. An integrated portable hand-held analyser for real-time isothermal nucleic acid amplification. *Anal. Chim. Acta* **598**, 286-294 (2007).
7 *Ahram Biosystems Inc.*, http://www.ahrambio.com/products_palmpcr_F1-12.html (2013).
8 HIV/AIDS Diagnostic Technology Landscape 3rd Edition. *UNITAID,* http://www.unitaid.org/images/marketdynamics/publications/UNITAID-HIV_Diagnostic_Landscape-3rd_edition.pdf (2013).
9 *IQuum*, http://www.iquum.com/products/analyzer.shtml (2013).
10 Apple iPhone 5 – Full phone specifications. *GSMArena*, http://www.gsmarena.com/apple_iphone_5-4910.php (2013).
11 Samsung I9500 Galaxy S4 - Full phone specifications. *GSMArena*, http://www.gsmarena.com/samsung_i9500_galaxy_s4-5125.php (2013).
12 Asus Google Nexus 7 - Full phone specifications. *GSMArena*, http://www.gsmarena.com/asus_google_nexus_7-4850.php (2013).
13 Cai, X. *et al.* Kaposi's sarcoma-associated herpesvirus expresses an array of viral microRNAs in latently infected cells. *P. Natl. Acad. Sci. U. S. A.* **102**, 5570-5575 (2005).
14 Altschul, S. F. *et al.* Gapped BLAST and PSI-BLAST: a new generation of protein database search programs. *Nucleic Acids Res.* **25**, 3389-3402 (1997).
15 Krishnan, M. & Erickson, D. Optically induced microfluidic reconfiguration. *Lab Chip* **12**, 613-621 (2012).
